# Effect of tyrosine kinase inhibitors on renal handling of creatinine by MATE1

**DOI:** 10.1038/s41598-018-27672-y

**Published:** 2018-06-18

**Authors:** Saki Omote, Natsumi Matsuoka, Hiroshi Arakawa, Takeo Nakanishi, Ikumi Tamai

**Affiliations:** Faculty of Pharmaceutical Science, Institute of Medical, Pharmaceutical and Health Sciences, Kanazawa University, Kakuma-machi, Kanazawa, 920-1192 Japan

## Abstract

Creatinine is actively secreted across tubular epithelial cells via organic cation transporter 2 (OCT2) and multidrug and toxin extrusion 1 (MATE1). We previously showed that the tyrosine kinase inhibitor (TKI) crizotinib inhibits OCT2-mediated transport of creatinine. In the present work, we examined the inhibitory potency of TKIs, including crizotinib, on MATE1-mediated transport of creatinine. Then, we used the kinetic parameters estimated in this and the previous work to predict the potential impact of TKIs on serum creatinine level (SCr) via reversible inhibition of creatinine transport. Crizotinib inhibited [^14^C]creatinine uptake by MATE1-overexpressing cells, and the inhibitory effect increased with incubation time, being greater in the case of pre-incubation or combined pre-incubation/co-incubation (pre/co-incubation) than in the case of co-incubation alone. The inhibition was non-competitive, with *K*_*i*_ values of 2.34 μM, 0.455 μM and 0.342 μM under co-, pre- or pre/co-incubation conditions, respectively. Similar values were obtained for inhibition of [^3^H]MPP^+^ uptake by MATE1-overexpressing cells. Gefitinib, imatinib, pazopanib, sorafenib, and sunitinib also inhibited MATE1-mediated creatinine uptake. Further, all these TKIs except pazopanib inhibited [^14^C]creatinine uptake by OCT2-overexpressing cells. In rat kidney slices, the ratio of unbound tissue accumulation of TKIs to extracellular concentration ranged from 2.05 to 3.93. Prediction of the influence of TKIs on SCr based on the renal creatinine clearance and plasma maximum unbound concentrations of TKIs suggested that crizotinib and imatinib might increase SCr by more than 10% in the clinical context. Accordingly, it is necessary to be cautious in diagnosing TKI-induced renal failure only on the basis of an increase of SCr.

## Introduction

Renal failure can be caused by various chemotherapeutic agents, and may require termination of the therapy or decrease of the dose. The Kidney Disease Improving Global Outcomes (KDIGO) clinical guidelines in 2012^1^ defined acute kidney injury (AKI) as: 1) an increase in the serum creatinine concentration (SCr) by more than ≧0.3 mg/dL within 48 hours; 2) an increase in SCr to ≧1.5 times baseline, which is known or presumed to have occurred within the prior 7 days; or 3) urine volume <0.5 ml/kg/h for 6 hours. In hospital practice, daily measurement of SCr is commonly used to monitor patients for AKI.

Creatinine is mainly excreted into urine by glomerular filtration and is partly secreted via transporters^2^, which account for 10–40% of total creatinine clearance, depending on kidney function^3^. Creatinine is a substrate of organic cation transporter 1 (OCT1), OCT2, organic anion transporter 1 (OAT1), OAT2, OAT3, multidrug and toxin extrusion (MATE1), and MATE2K^4–7^. Genome-wide association studies indicate that genetic mutations of OCT2 and MATE1 affect creatinine clearance or SCr^8,9^, and OCT2 and MATE1 are expressed at the basolateral and apical membranes of renal tubular epithelial cells, respectively^4,6^. These creatinine transporters are inhibited by various drugs, including cimetidine, DX-619, pyrimethamine, and trimethoprim^2,10^. A physiologically based pharmacokinetic (PBPK) model analysis by Imamura *et al*. indicated that DX-619, an antibacterial agent, could reduce creatinine clearance by up to 51.4%, by decreasing the creatinine secretion mediated by these transporters^10^. Such an increase of SCr due to inhibition of creatinine transporters by drugs might lead to incorrect diagnosis of drug-induced renal failure. It is already known that TKIs such as crizotinib, gefitinib, imatinib, pazopanib, sorafenib, and sunitinib clinically increase SCr^11–16^, and have the potential to inhibit OCT2 and/or MATE1^17–20^. Therefore, the increase of SCr by TKIs could be due to either true renal failure or reversible interaction with creatinine transporters. Thus, it is clinically important to evaluate the inhibitory potential of TKIs towards creatinine transporters in order to manage chemotherapy appropriately.

We recently reported that crizotinib is an inhibitor of OCT2-mediated uptake of creatinine, and we found that its inhibitory effect after pre-incubation or combined pre-incubation/co-incubation (pre/co-incubation) was greater than that in the case of co-incubation alone^20^. Furthermore, the inhibitory effect of crizotinib on OCT2 was substrate-dependent. It is important to know whether inhibition of MATE1, which mediates renal secretion of creatinine at the apical membrane, by TKIs shows similar characteristics. Therefore, in the present study we examined on the inhibitory effects of TKIs, including crizotinib, on MATE1-mediated uptake of creatinine. Furthermore, we used the kinetic parameters obtained in the present and previous work to model the effect of TKI-induced inhibition of creatinine transporters on SCr in order to assess whether it might be significant in relation to the clinical diagnosis of TKI-induced renal failure.

## Results

### Inhibitory Effect of Crizotinib on Creatinine and MPP^+^ Uptake by MATE1-Overexpressing HEK293 Cells

**Figure 1:**
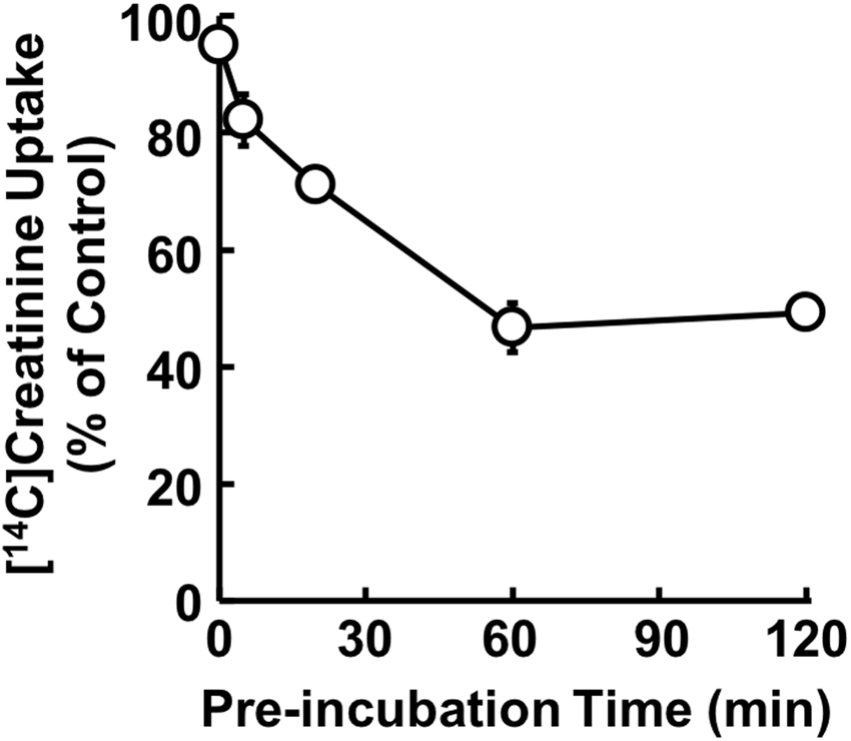
Time dependence of the inhibitory effect of crizotinib on [^14^C]creatinine uptake by HEK293/MATE1 cells. Uptake of [^14^C]creatinine (2.6 μM) by HEK293/MATE1 cells was measured for 2 min in the presence of 2 μM crizotinib after pre-incubation with crizotinib for 0, 5, 20, 60, and 120 min. Data are shown as percent of the control measured in the absence of crizotinib. Bars indicate ± S.E.M. (n = 3), and if not shown, are smaller than the symbol.

**Figure 2:**
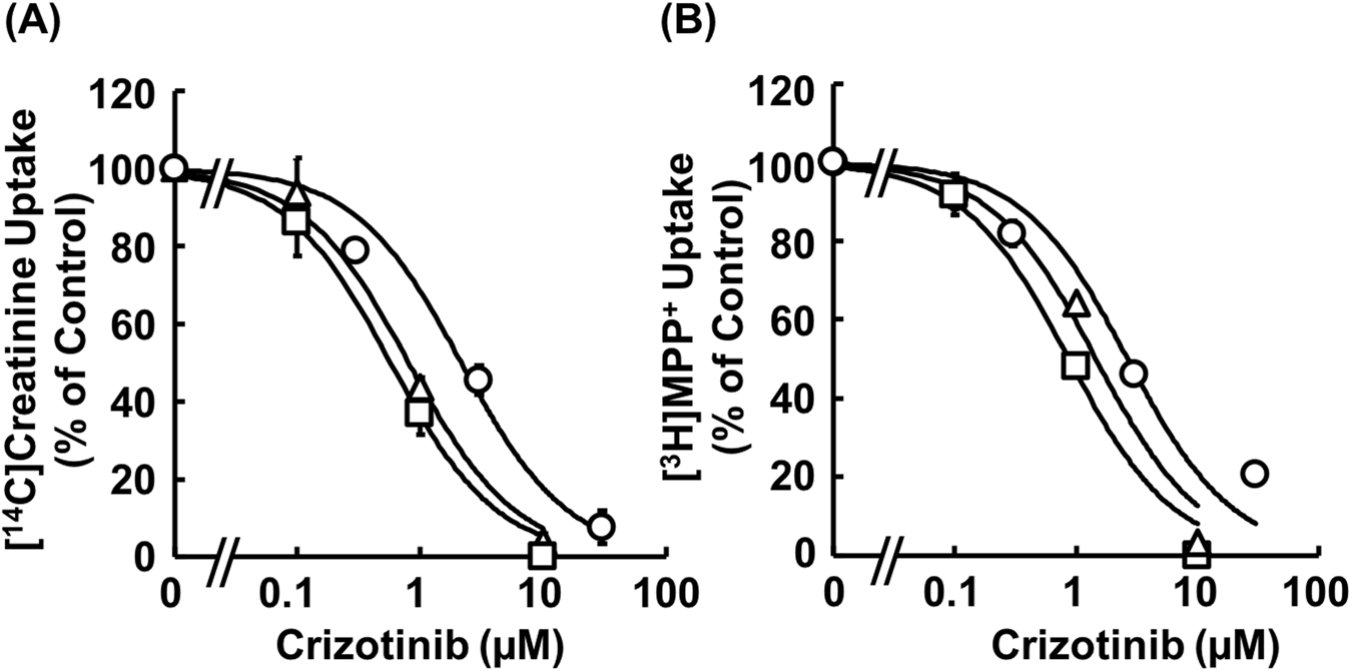
Inhibitory effect of crizotinib on [^14^C]creatinine and [^3^H]MPP^+^ uptake by HEK293/MATE1 cells. The cells were pre-incubated with or without crizotinib at the indicated comcentration for 60 min, and then MATE1-mediated uptake of (**A**) [^14^C]creatinine (2.5 μM) or (**B**) [^3^H]MPP^+^ (2.5 nM) were measured in the absence or the presence of crizotinib for 2 min. Co-, pre- or pre/co-incubation of crizotinib is indicated by circles, triangles or squares, respectively. Bars indicate ± S.E.M. (n = 3), and if not shown, are smaller than the symbol.

Uptake of [^14^C]creatinine by HEK293/MATE1 cells was significantly higher than that by mock cells, and since the uptake increased almost linearly up to 3 min (Supplemental Fig. 1A), initial uptakes were evaluated at 2 min in subsequent experiments. On the other hand, although uptake of [^14^C]creatinine by HEK293/MATE2K cells was comparable to that by mock cells, clear uptake of MPP^+^ was observed (mock: 5.76 μL/mg/1 min; HEK293/MATE2K: 19.1 μL/mg/1 min). Then, we examined the time dependence of inhibition of MATE1-mediated [^14^C]creatinine uptake by crizotinib. We found that the extent of the inhibition increased time-dependently, reaching a plateau after 60 min pre-incubation (Fig. 1A). Therefore, the pre-incubation time was set at 60 min in subsequent studies. Next, the inhibitory effect was compared among co-incubation, pre-incubation, and pre/co-incubation protocols at a fixed substrate concentration. Co-, pre-, and pre/co-incubation with crizotinib concentration-dependently reduced the uptake of [^14^C]creatinine by HEK293/MATE1 cells with *IC*_*50*_ values of 2.16 ± 0.31 μM, 0.804 ± 0.132 μM, and 0.573 ± 0.102 μM, respectively (Fig. 2A). Similarly, co-, pre-, and pre/co-incubation with crizotinib reduced the uptake of [^3^H]MPP^+^ by HEK293/MATE1 cells with *IC*_*50*_ values of 2.66 ± 0.46 μM, 1.45 ± 0.22 μM, and 0.868 ± 0.097 μM, respectively (Fig. 2B). On the other hand, MATE2K-mediated [^3^H]MPP^+^ uptake was inhibited by crizotinib with *IC*_*50*_ values in the range of 8.8 ± 3.4 μM (Supplemental Fig. 1B), showing that the inhibitory potency of crizotinib on MATE2K was less than that on MATE1. Accordingly, in the present study, we considered that TKIs would predominantly influence MATE1-mediated creatinine secretion, and that the effect on MATE2K could be neglected.

### Kinetic Analysis of the Inhibitory Effect of Crizotinib on Creatinine Uptake by MATE1-Overexpressing HEK293 Cells

**Figure 3:**
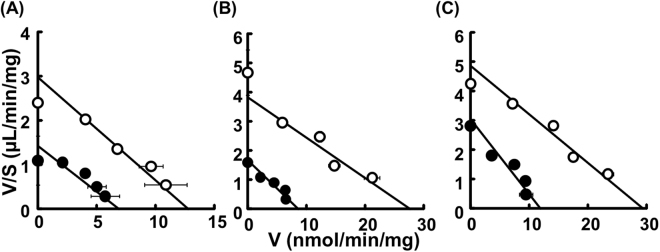
Kinetic analysis of crizotinib inhibition of creatinine uptake by HEK293/MATE1 cells. The effect of (**A**) co-, (**B**) pre-, (**C**) pre/co-incubation effect of crizotinib on MATE-mediated uptake of [^14^C]creatinine (3.9 μM) was examined. The cells were pre-incubated with or without crizotinib for 60 min, and then MATE1-mediated uptake of creatinine was measured at various concentrations in the absence (open circles) or the presence (closed circles) of crizotinib for 2 min. Bars indicate ± S.E.M. (n = 3 or 4), and if not shown, are smaller than the symbol.

To study the inhibitory mechanism of crizotinib on MATE1-mediated [^14^C]creatinine uptake, the concentration dependence of creatinine uptake by HEK293/MATE1 cells in the presence and absence of crizotinib was examined (Fig. 3A–C). The results are summarized in Table 1. Under all treatment conditions (co-, pre-incubation and pre/co-incubation), crizotinib reduced the apparent *V*_*max*_ value, while the *K*_*m*_ value was essentially unaffected, demonstrating that crizotinib is a non-competitive inhibitor of creatinine uptake by MATE1 under all these incubation conditions. The *K*_*i*_ values were 2.34 μM, 0.455 μM, and 0.342 μM under co-, pre- or pre/co-incubation conditions, respectively.

**Table 1:** Kinetic parameters (*K*_*m*_*, V*_*max*_ and *K*_*i*_) of creatinine uptake by MATE1 under various assay conditions.

Assay Conditions	*K*_*m*_ (mM)	*V*_*max*_ (nmol/min/mg)	*K*_*i*_ (μM)
Control	4.31 ± 1.21	12.8 ± 1.4	—
Co-incubation with 2 μM crizotinib	4.86 ± 1.96	6.89 ± 1.15	2.34
Control	7.26 ± 1.29	27.7 ± 2.3	—
Pre-incubation with 1 μM crizotinib	5.21 ± 1.14	8.66 ± 0.80	0.455
Control	6.06 ± 1.00	29.4 ± 2.2	—
Pre/co-incubation with 0.5 μM crizotinib	3.91 ± 0.87	11.9 ± 1.0	0.342

Data are mean ± S.D.

### Intracellular Accumulation of Crizotinib during Pre-incubation

**Figure 4:**
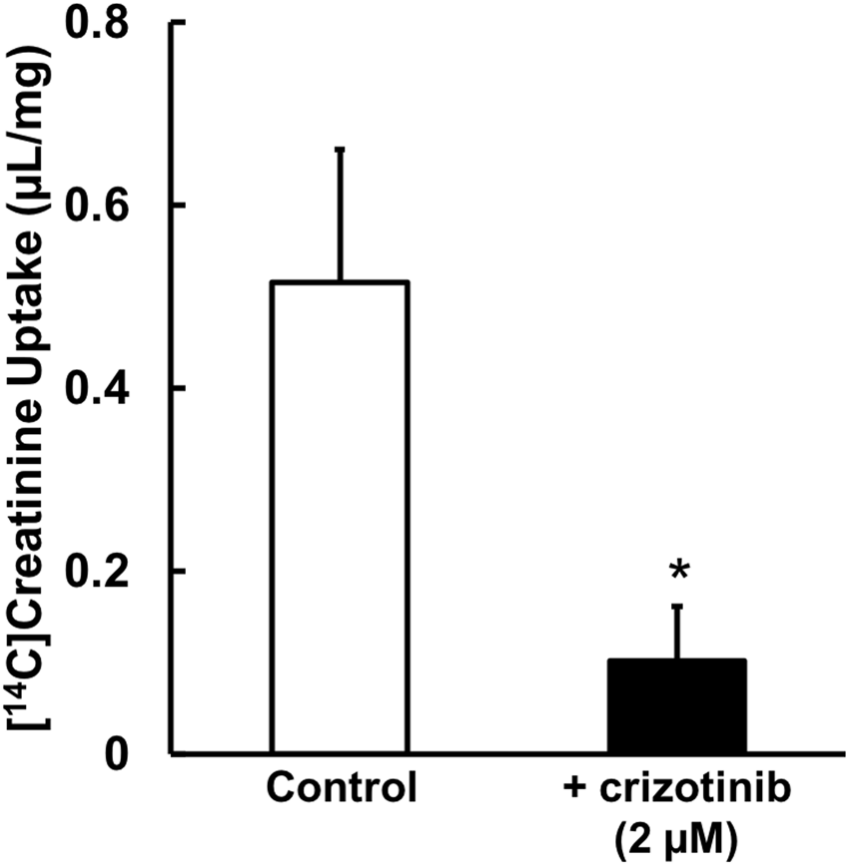
Time-dependent uptake of [^14^C]creatinine uptake by MDCKII/MATE1 cells. The cells were pre-incubated with or without (control) 2 μM crizotinib for 60 min, and then MATE1-mediated uptake of [^14^C]creatinine (3.8 μM) was measured for 2 min. Columns show the mean, and bars indicate + S.E.M (n = 3). *Indicates a significant difference from the control (*p* < 0.05) by Student’s *t*-test.

We hypothesized that the time dependence of the inhibition (pre-incubation effect) is due to intracellular accumulation of crizotinib. Accordingly, to test this idea, we examined the intracellular accumulation of crizotinib using monolayers of MDCKII/MATE1 cells in a Transwell system. Pre-incubation of the cells with crizotinib (2 μM) added on the basolateral side alone reduced [^14^C]creatinine uptake from the apical side (Fig. 4). In this experiment, the observed crizotinib concentration in the apical side medium after the pre-incubation was 0.105 ± 0.015 μM, which is below the *Ki* value under the co-incubation condition. Accordingly, since the observed reduction of creatinine uptake from the apical side cannot be accounted for by the crizotinib concentration in the apical side medium, these findings confirm that the pre-incubation effect is due to the intracellular accumulation of crizotinib.

### Inhibitory Effects of Other TKIs on Creatinine Uptake by OCT2- and MATE1-Overexpressing HEK293 Cells and TKI accumulation in Rat Kidney Slices

**Figure 5:**
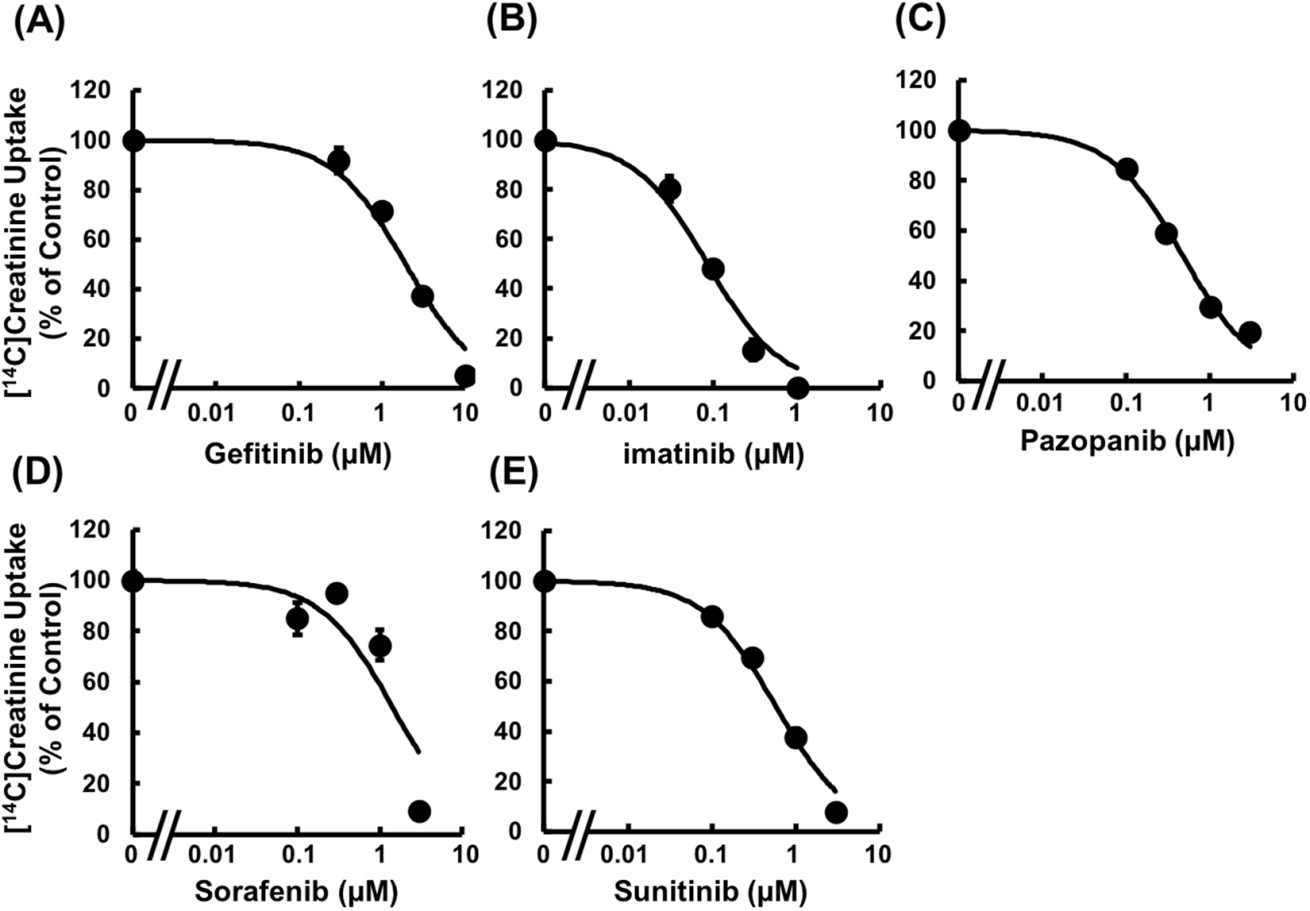
Concentration dependence of the inhibitory effect of TKIs on creatinine uptake by HEK293/MATE1 cells. Uptake of [^14^C]creatinine (2.6 μM) by HEK293/MATE1 cells was measured at 37 °C for 2 min in the absence or presence of (**A**) gefitinib (0.3, 1, 3, and 10 μM), (**B**) imatinib (0.03, 0.1, 0.3, and 1 μM), (**C**) pazopanib (0.1, 0.3, 1, and 3 μM), (**D**) sorafenib (0.1, 0.3, 1, and 3 μM), and (**E**) sunitinib (0.1, 0.3, 1, and 3 μM). Each point indicates the mean of 3 determinations. Bars indicate ± S.E.M. (n = 3), and if not shown, are smaller than the symbol.

**Figure 6:**
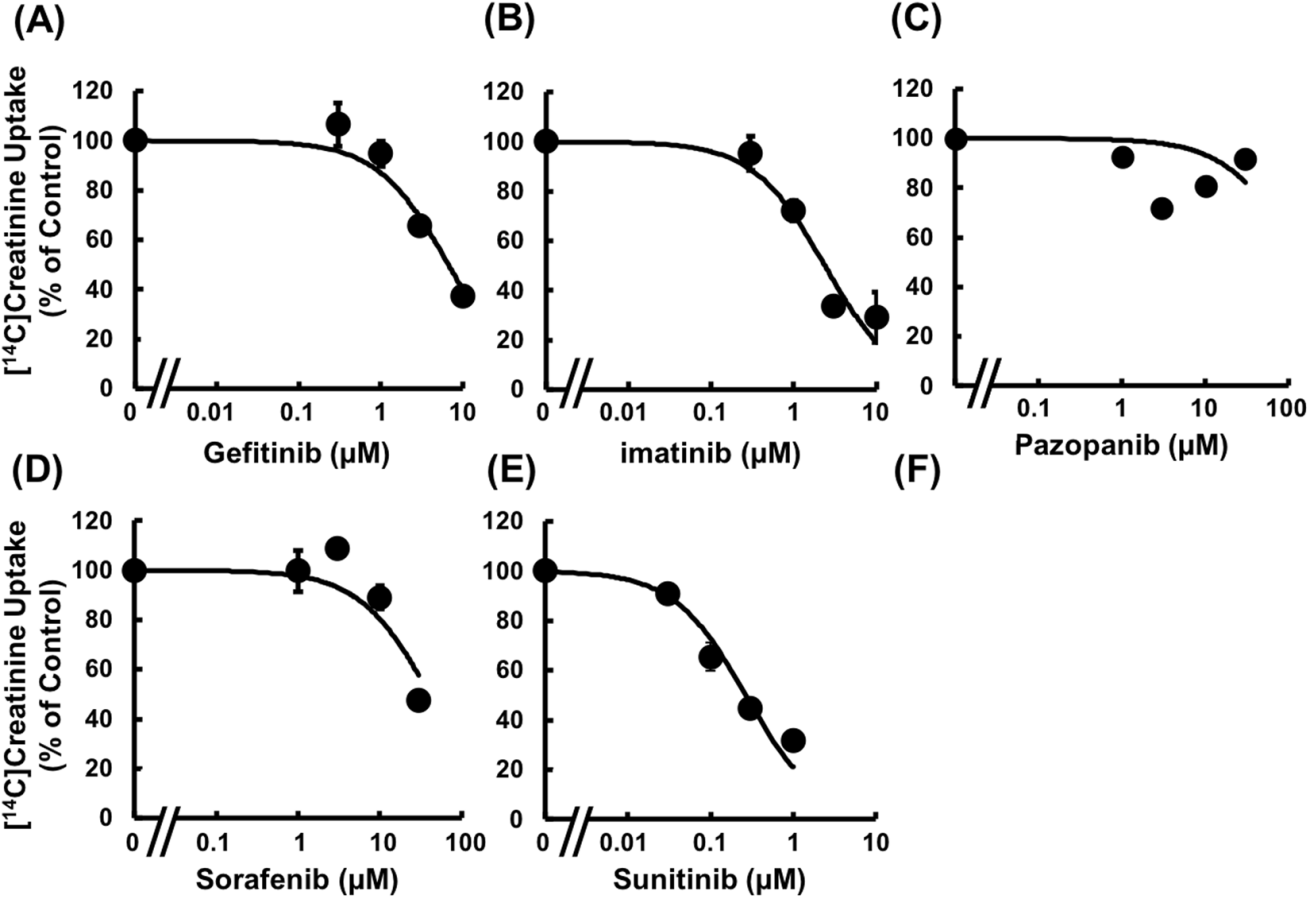
Concentration dependence of the inhibitory effect of TKIs on creatinine uptake by HEK293/OCT2 cells. Uptake of [^14^C]creatinine (2.6 μM) by HEK293/OCT2 cells was performed at 37 °C for 1 min in the absence or presence of (**A**) gefitinib (0.3, 1, 3, and 10 μM), (**B**) imatinib (0.3, 1, 3, and 10 μM), (**C**) pazopanib (1, 3, 10, and 30 μM), (**D**) sorafenib (1, 3, 10, and 30 μM), and (**E**) sunitinib (0.03, 0.1, 0.3, and 1 μM). Each point indicates the mean of 3 determinations. Bars indicate ± S.E.M. (n = 3), and if not shown, are smaller than the symbol.

To study the inhibitory potential of other TKIs on OCT2- and MATE1-mediated uptake of [^14^C]creatinine, the concentration dependence of creatinine uptake by HEK293/MATE1 and HEK293/OCT2 cells was examined in the presence of several TKIs. Firstly, in order to confirm that the pre-incubation effect is not specific to crizotinib, the effects of co- and pre/co-incubation with imatinib on [^14^C]creatinine uptake by HEK293/MATE1 cells were examined. Imatinib reduced [^14^C]creatinine uptake with *IC*_*50*_ values of 0.466 ± 0.053 μM and 0.0651 ± 0.0087 μM under co- and pre/co-incubation conditions, respectively (Supplemental Fig. 2), demonstrating that the pre-incubation effect is not specific to crizotinib. Since pre-incubation is considered to be clinically relevant and results in greater inhibitory potency, the pre/co-incubation condition was used to examine the effects of other TKIs. As shown in Fig. 5, [^14^C]creatinine uptake by HEK293/MATE1 cells was decreased in the presence of each of imatinib, gefitinib, pazopanib, sunitinib, and sorafenib in a concentration-dependent manner. The order of inhibitory potency for MATE-mediated uptake of [^14^C]creatinine was imatinib > crizotinib > pazopanib > sunitinib > sorafenib > gefitinib. In addition, [^14^C]creatinine uptake by HEK293/OCT2 cells was inhibited by imatinib, gefitinib, sunitinib, and sorafenib, though not by pazopanib (Fig. 6), and the order of inhibitory potency of TKIs for OCT2-mediated uptake of [^14^C]creatinine was sunitinib > crizotinib > imatinib > gefitinib > sorafenib > pazopanib. The *IC*_*50*_ values of TKIs for OCT2- and MATE1-mediated uptake of creatinine are summarized in Table 2.

**Table 2:** Pharmacokinetic parameters and obtained *IC*_*50*_, *K*_*i*_, and *K*_*p,uu*_ values of TKIs.

Drugs	C_max_ (μM) C_trough_ (μM)	fu	C_max,u_ (μM) C_trough,u_ (μM)	*t*_*1/2*_ (hr)	*IC*_*50*_ or *K*_*i*_		*C*_max,u_ or *C*_trough_*/IC*_*50*_ o*r K*_*i*_		K_p,uu_	References for clinical parameters
					OCT2	MATE1	OCT2	MATE1		
Crizotinib	0.912	0.093	0.0848	42	0.338^a^	0.342	0.251	0.248	3.28 ± 0.28	^20,34^
	0.708		0.0658				0.195	0.193		
Gefitinib	1.04	0.09	0.0936	23.8	6.68 ± 1.10	1.92 ± 0.23	0.0140	0.0488	2.22 ± 0.05	^35^
	—		—				—	—		
Imatinib	5.80	0.048	0.278	19.3	2.37 ± 0.43	0.0857 ± 0.0102	0.116	3.24	2.39 ± 0.29	^36,37^
	2.72		0.130				0.0543	1.52		
Pazopanib	95.1	0.000106	0.0101	30.9	—	0.470 ± 0.032	0.0000725	0.0214	2.05 ± 0.14	^38,39^
	50.6		0.0536				0.0000386	0.0114		
Sorafenib	14.6	0.0029	0.0423	28.1	40.8 ± 9.3	1.43 ± 0.36	0.00104	0.0296	2.76 ± 0.27	^40,41^
	—		—				—	—		
Sunitinib	0.136	0.05	0.00680	40–60	0.266 ± 0.030	0.582 ± 0.051	0.0256	0.0117	3.93 ± 0.15	^42,43^
	0.0829		0.00414				0.0153	0.00714		

*K*_*p,uu*_ values are mean ± S.E.M. (n = 3 or 4). Dosing regimen; crizotinib: 250 mg twice a day, gefitinib: 250 mg once a day, imatinib: 400 mg once a day, pazopanib: 800 mg once a day, sorafenib: 400 mg once a daily, sunitinib: 50 mg once a daily.

C_max_, maximum plasma concentration; f_u_, unbound fraction in plasma; C_max, u_, calculated unbound plasma concentration. The clinical parameters were obtained from the cited references.

^a^The values were obtained from Arakawa *et al*.^20^.

**Figure 7:**
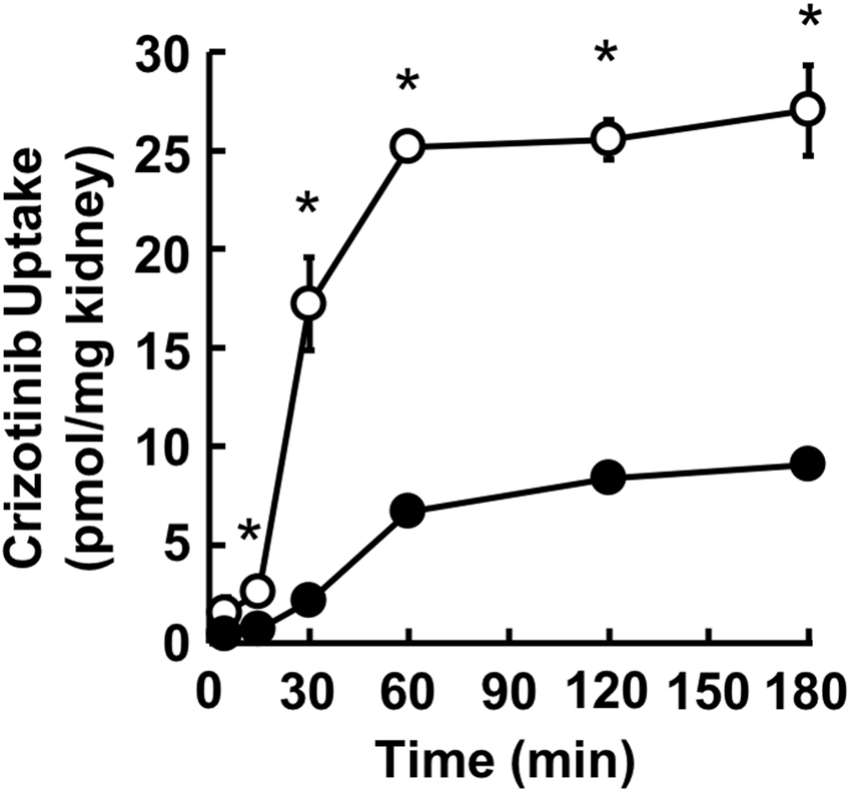
Time-dependent uptake of crizotinib by rat kidney slices. Uptake of crizotinib (1 μM) was measured at 37 °C or 4 °C for 5, 15, 30, 60, 120 and 180 min. Closed and open circles represent creatinine uptake at 37 °C and 4 °C, respectively. Each point indicates the mean of 3 determinations. Bars indicate ± S.E.M. (n = 3), and if not shown are smaller than the symbol. *Indicates a significant difference from the uptake at 4 °C and that at 37 °C at the same time point (*p* < 0.05) by Student’s *t*-test.

We also examined the tissue accumulation of crizotinib in rat kidney slices. The uptake of crizotinib at 37 °C was markedly higher than that at 4 °C (Fig. 7), suggesting carrier-mediated uptake of crizotinib into rat kidney slices. Because the uptake attained a steady-state at 60 min, the *K*_*p,uu*_ values of TKIs were calculated at 120 min. The *K*_*p,uu*_ value of crizotinib was 3.28 ± 0.28, which suggests that crizotinib is concentratively accumulated in proximal tubular cells. The *K*_*p,uu*_ values of gefitinib, imatinib, pazopanib, sorafenib, and sunitinib were also determined, and ranged from 2.05 ± 0.28 (pazopanib) to 3.93 ± 0.15 (sunitinib), as shown in Table 2.

The determined *IC*_*50*_ and *K*_*p,uu*_ values were used, in conjunction with parameters taken from a previous clinical study of 11 subjects^10^, to predict the increment of SCr due to inhibition of transporter-mediated creatinine uptake by TKIs. As shown in Table 3, the results indicate that crizotinib and imatinib could cause an increase of SCr of more than 10% in the clinical context.

**Table 3:** Prediction of clinical increase of SCr.

Drugs	Prediction of SCr increase (%)			
	C_max,u_	C_trough,u_	C_max,u_ · *K*_*p,uu*_	C_trough,u_ · *K*_*p,uu*_
Crizotinib	14.5 ± 4.6	11.7 ± 3.6	25.3 ± 8.8	21.1 ± 7.1
Gefitinib	2.47 ± 1.05	—	4.27 ± 1.43	—
Imatinib	42.0 ± 16.9	29.4 ± 10.6	52.6 ± 23.0	42.6 ± 17.2
Pazopanib	1.16 ± 0.87	0.837 ± 0.842	1.86 ± 0.95	1.22 ± 0.87
Sorafenib	1.47 ± 0.90	—	3.10 ± 1.17	—
Sunitinib	1.65 ± 0.92	1.19 ± 0.87	2.73 ± 1.10	1.86 ± 0.95

Data are mean ± S.D. (n = 11). Individual parameters of 11 subjects were taken from ref.^10^.

## Discussion

Most TKIs have the potential to cause renal failure, and since SCr is a major indicator of acute and chronic renal failure, it is clinically important to understand the potential of these drugs to cause reversible inhibition of renal creatinine transporters in order to avoid incorrect diagnosis of drug-induced renal failure. We previously showed that crizotinib in the clinically relevant concentration range has the potential to inhibit OCT2-mediated creatinine transport^20^. In the present study, therefore, we focused on the potential of TKIs, including crizotinib, to inhibit uptake of creatinine by MATE1, the other major renal creatinine transporter. Moreover, we aimed to use the estimated kinetic parameters to predict the extent of SCr increase that might be caused by TKIs through inhibition of transporter-mediated uptake of creatinine.

Firstly, we considered the effect of pre-incubation on the inhibitory potential of crizotinib for MATE1-mediated transport of creatinine, because kidney tissues are clinically exposed to administered drugs for a long period of time and many previous studies have been done without pre-incubation. As shown in Fig. 1, crizotinib inhibited MATE1-mediated uptake of [^14^C]creatinine in a time-dependent manner, as we had previously found in the case of crizotinib inhibition of OCT2^20^. Accordingly, pre-incubation for an appropriate period is essential for adequate evaluation of the inhibitory activity of drugs towards creatinine transport by OCT2 and MATE1. Secondly, many drugs may be transported by OCT2 and/or MATE1 to various extents^21–25^, so we examined whether the inhibitory effects of TKIs on MATE1 are substrate-dependent. In this context, we previously reported that the *IC*_*50*_ value of crizotinib for MPP^+^ uptake by OCT2 was 10–20 times higher than that for creatinine uptake^20^. Here, however, in contrast to the case of OCT2, we found that the *IC*_*50*_ values of crizotinib for MATE1 were comparable with creatinine and MPP^+^ as substrates (Fig. 2). This apparent discrepancy may be due to different inhibition mechanisms; crizotinib appears to be a competitive inhibitor of OCT2, but a non-competitive inhibitor of MATE1 (Fig. 3). The mechanism of the the crizotinib-mediated reduction of *V*_*max*_ value of creatinine uptake by MATE1 is uncertain. One possibility would be a decrease of MATE1 protein in the cellular membrane due to the altered trafficking, but this seems unlikely, since our preliminary study showed no change of intracellular signals of green fluorescent protein (GFP)-fused MATE1 protein in response to crizotinib. Accordingly, other mechanisms such as structural changes of MATE1 due to an allosteric effect of bound crizotinib, or dephosphorylation or degradation of MATE1, which could be associated with the pharmacological effect of crizotinib, should be considered. It has been reported that another TKI, dasatinib, reduced OCT2 activity by inhibiting the Src family kinase YES1, resulting in dephosphorylation of OCT2^19^. Therefore, the mechanism by which crizotinib inhibits MATE1 might involve dephosphorylation of MATE1 via inhibition of an unknown kinase(s). In addition, we found that creatinine was not transported by MATE2K in the present study, contrary to a previous report^6^. This apparent discrepancy might be explained by differences in the amounts of MATE2K expressed in the two models. In any event, crizotinib was a markedly less potent inhibitor of MATE2K than MATE1. Therefore, MATE2K was not incorporated in the equation for prediction of creatinine clearance, as its contribution to the effect of TKIs is presumed to be small.

Next, to aid in predicting the inhibitory potential of TKIs on MATE1 in the clinical context, we estimated the intracellular concentrations of TKIs, because MATE1 excretes intracellular creatinine to the tubular lumen. Tissue accumulation, *K*_*p,uu*_, of crizotinib in kidney was estimated using rat kidney slices. For concentration setting, it is preferable to use the clinically relevant unbound concentration. However, the clinically unbound concentration of some TKIs is as low as about 10 nM. It is technically infeasible to use such low concentrations experimentally, because TKIs are extensively adsorbed on the experimental apparatus; therefore, the concentration of TKIs was unified to 1 μM in this study. *In vitro* uptake of crizotinib by the kidney slices showed a significant temperature dependence, suggesting that it is transporter-mediated, at least in part. Although the exact mechanism of crizotinib uptake into proximal tubular epithelial cells is unclear, it has been reported that several TKIs are substrates of solute carrier transporters, including OCT2^26^. Thus, transporters expressed in tubular epithelial cells, such as OCT2 and organic anion transporter 3 (OAT3), may play a role in accumulation of TKIs in kidney. Nevertheless, it will be important in the future to establish the mechanism of the pre-incubation effect in order to ensure that the prediction of SCr change is soundly based.

Finally, we used the estimated kinetic parameters in a modeling analysis to investigate whether inhibition of transporters by TKIs might cause a clinically significant increase of SCr. As shown in Table 3, based on the renal creatinine clearance equation^10^ and the reported C_max,u_, crizotinib could increase SCr by 14.5%, and the increment is increased to 25.3% when the *K*_*p,uu*_ value is taken into consideration. We did not incorporate MATE2K in the equation for prediction of SCr, because creatinine was negligibly transported by HEK293/MATE2K cells, and also because crizotinib was a much less potent inhibitor of MATE2K, compared with OCT2 and MATE1. Moreover, the intrinsic clearances for OCT2 and MATE1 are similar, since the protein amounts of OCT2 and MATE1 are comparable in human kidney^27^, and a previous study well predicted the clinical increase of SCr based on this assumption^10^. The clinically reported eGFR reduction in patients treated with crizotinib was 23.9% (n = 38, 95% confidence interval (CI): 21.3–26.6%)^28^. Bearing in mind that crizotinib has a long half-life (t_1/2_) of 42 hr, the predicted increase of SCr estimated from C_trough,u_ might also be consistent with the clinical observation. Our modeling analysis also showed that imatinib could increase SCr by 42.0% and 29.4% based on C_max,u_ and C_trough,u_, respectively, and these values increased to 52.6% and 42.6%, respectively, after incorporation of *K*_*p,uu*_. Again, these values are broadly consistent with the clinically reported decrease of eGFR of 27.1% (mean value calculated from 7 subjects^29^). Considering a report that increasing effect of imatinib on SCr was reversible^13^, our conclusion here regarding imatinib appears to be clinically relevant. These results support the idea that specific interaction of some TKIs with renal creatinine transporters may lead to a reversible increase of SCr in the absence of renal failure. However, since it is also possible that crizotinib and imatinib cause true adverse events in kidney, we emphasize that it is desirable to use not only SCr, but also additional renal function markers such as blood urea nitrogen, to distinguish between reversible and true drug-induced renal failure. Moreover, it should be noted that incorporation of *K*_*p,uu*_ values in the prediction may or may not be necessary depending on the mechanism of the pre-incubation effect; it is necessary if the pre-incubation effect is caused by kinase inhibition, but not if the pre-incubation effect is determined by the intracellular concentration of drugs. Further study will be needed to clarify the mechanism in order to improve the prediction of the changes in creatinine disposition.

In conclusion, the present study demonstrated that TKIs such as crizotinib and imatinib have the potential to inhibit creatinine transport via MATE1, as well as OCT2, at clinically relevant concentrations. The resulting reversible increase of SCr could lead to incorrect diagnosis of drug-induced renal failure.

## Materials and Methods

### Materials

[^14^C]Creatinine (58 mCi/mmol) and [^3^H]N-methyl-4-phenylpyridinium acetate (MPP^+^, 80 Ci/mmol) were purchased from Moravek Biochemicals (Brea, CA) and American Radiolabeled Chemicals, Inc. (St. Louis, MO), respectively. Non-labeled creatinine and crizotinib were purchased from Wako Pure Chemical Industries, Ltd., (Osaka, Japan) and AdooQ Bioscience, LLC (Irvine, CA), respectively. All other chemicals were commercial products of reagent grade. pCMV-SPORT6/MATE1 (clone name: IRAK013O20) was obtained from RIKEN BRC (the National Bio-Resource Project of the MEXT, Japan).

### Animals

Male Wistar rats (180 ± 10 g body weight) were purchased from Japan SLC (Hamamatsu, Japan). Rats were housed three per cage with free access to commercial chow and tap water, and were maintained on a 12 h dark/light cycle in an air-controlled room (temperature, 24.0 ± 1 °C; humidity, 55 ± 5%). All animal studies were approved by the Kanazawa University Institutional Animal Care and Use Committee (Permit number, AP-163750), and were performed in accordance with the university guidelines.

### Cell Culture

HEK293 cells transfected with human *OCT2* cDNA (HEK293/OCT2) or vector alone (mock) were prepared in our laboratory as described previously^30^. HEK293 cells transfected with human MATE1 (HEK293/MATE1) and vector alone (mock) were gifts from Dr. Inoue (Tokyo University of Pharmacy and Life Science). To prepare MATE1-overexpressing MDCKII cells (MDCKII/MATE1), MDCKII cells were similarly transfected with human *MATE1* cDNA (see below). Cells were cultured in Dulbecco’s modified Eagle’s medium (DMEM) supplemented with 10% fetal bovine serum, 100 units/ml penicillin, 100 μg/ml streptomycin, 1 mM pyruvic acid, and 100 μg/ml zeocin for HEK293/OCT2 cells or 400 μg/mL G418 for HEK293/MATE1 and MDCKII/MATE1 cells at 37 °C in a humidified atmosphere of 5% CO_2_ in air.

### Construction of the Expression Plasmid for MATE1

The complete cDNA sequence of human MATE1 was amplified by PCR using specific oligonucleotides with additional restriction enzyme sites as follows: sense (with BamHI site), 5′-TACCGAGCTCGGATCAGTCACATGGAAGCTCCTGA-3′ and antisense (with BamHI site), 5′-CTGGACTAGTGGATCCACGTCACTGAATTCTGACATAG-3′ from pCMV-SPORT6/MATE1. The sequence was verified with a DNA sequencer (ABI PRISMTM 310 Genetic Analyzer, Applied Biosystems). The MATE1 coding region was inserted in pcDNA3.1 to obtain MATE1-plasmid (pcDNA3.1/MATE1).

### Uptake by Transporter-expressing Cells

The uptake experiment was conducted as described previously^20^. HEK293 cells were plated at a density of 1.7 × 10^5^ cells/cm^2^ on 24-well plates and cultured for two days before uptake assay. The cells were pre-incubated with 0.5 ml of transport medium (TM) (125 mM NaCl, 4.8 mM KCl, 1.2 mM KH_2_PO_4_, 1.2 mM CaCl_2_, 1.2 mM MgSO_4_, 5.6 mM D-glucose, and 25 mM HEPES, pH 7.4) for an appropriate time at 37 °C. Cells for MATE1 assays were pre-incubated in TM containing 30 mM NH_4_Cl for 20 min to establish an outwardly directed H^+^ gradient; this was done concomitantly with the pre-incubation with TKIs. To measure co-, pre- or both (pre/co-) incubation effects of TKIs, the cultured cells were pre-incubated with TM in the absence or presence of TKIs for predetermined periods, and uptake was initiated by replacing 0.25 ml of TM with TM containing [^14^C]creatinine or [^3^H]MPP^+^ in the absence or the presence of TKIs. The cells were incubated at 37 °C. Uptake was terminated by washing the cells three times with 0.5 ml of ice-cold TM, and the cells were solubilized in 0.25 ml of 0.01% (v/v) Triton-X. Radioactivity was measured with a liquid scintillation counter (Hitachi Aloka Medical, Ltd., Tokyo, Japan). Part of the lysate was used for the determination of total protein amount with a protein assay kit (Bio-Rad Laboratories, Hercules, CA). For uptake assay using the Transwell system, MDCKII cells were plated at a density of 2.2 × 10^5^ cells/cm^2^ on a 12-well Transwell filter insert for 5 days before uptake study. After adding TM containing 2 μM crizotinib for 60 min, the medium on both sides was removed and the cells were washed three times with ice-cold TM. Uptake study was initiated by adding TM containing [^14^C]creatinine and TM alone on the apical and basolateral sides, respectively. Uptake was terminated by washing the cells three times with ice-cold TM, and intracellular [^14^C]creatinine concentration was determined by the method described above. In the experiments with crizotinib, non-labeled creatinine was used and the concentration was determined by LC-MS/MS. After initiation of uptake, the medium on the apical side was collected at 2 min, and the crizotinib concentration was determined.

### Uptake by Rat Kidney Slices

Uptake study by rat kidney slices was carried out as described previously, with slight modifications^31^. Whole kidneys of male rats were sliced (0.3 mm thick) with a microslicer (Zero 1; Dosaka EM, Kyoto, Japan). The slices were immediately put in ice-cold oxygenated transport buffer (130 mM NaCl, 4.8 mM KCl, 1.2 mM CaCl_2_, 1.2 mM MgSO_4_, 1.2 mM KH_2_PO_4_ and 25 mM HEPES, adjusted to pH 7.4). Three or four slices per rat, each weighing 5 to 20 mg, were randomly selected and pre-incubated in a 12-well plate with 2.0 ml/well of oxygenated transport buffer at 37 °C for 5 min. After pre-incubation, the kidney slices were placed in transport buffer containing TKIs to initiate the uptake reaction. After uptake at 37 °C for an appropriate time, each slice was rapidly removed from the transport buffer, washed twice in ice-cold transport buffer, blotted on filter paper and weighed. Non-specific adsorption of each substrate was estimated as the absorption on a slice incubated at 4 °C for 1 min. The tissue accumulation of TKIs was measured by liquid chromatography-tandem mass spectrometry (LC-MS/MS) as described below.

### Measurement of TKIs by LC-MS/MS

The amounts of TKIs were determined with a LCMS-8050 triple quadrupole LC-MS/MS (Shimadzu, Kyoto, Japan) coupled to an LC-30A system (Shimadzu) using an ACQUITY UPLC BEH C18 column (130 Å, 1.7 μm, ID 2.1 mm × 50 mm; Waters Corporation, MA, USA) at 40 °C. The mobile phase was composed of a mixture of 0.1% formic acid in water (pH 3.0) and 0.1% formic acid in acetonitrile at the flow rate of 0.2 mL/min. The mass numbers of the molecular and product ions for each compound were as follows: crizotinib (449.9 → 260.2, CE −24 V), gefitinib (447.2 → 128.2, CE −23 V), imatinib (494.3 → 394.3, CE −28 V) pazopanib (438.1 → 357.2, CE −35 V), sorafenib (464.9 → 252.0, CE −21 V), and sunitinib (399.2 → 283.0, CE −29 V). Labsolutions software (version 5.89, Shimadzu) was used for data manipulation. The detection limit was 10 nM for each compound.

### Data analysis

Experiments were conducted at least twice, and the results are shown as representative values in individual experiments. Uptake by transporter-expressing cells was expressed as the cell-to-medium (C/M) ratio (μL/mg protein), obtained by dividing the uptake amount by the concentration of substrate in the TM. MATE1- or OCT2-mediated uptake was obtained after subtraction of the uptake by mock cells.

Kinetic parameters were calculated by means of a nonlinear least-squares method using KaleidaGraph (Synergy Software, Reading, PA), and are shown as mean +/− statistical errors from a single experiment. The inhibitory effect of TKIs was expressed as a percentage of the control, and the *IC*_*50*_ value was obtained by means of the following Eq. (1);

1 $$ \% \,of\,control=\frac{100\times I{C}_{50}}{I{C}_{50}+[I]}$$

where [I] is inhibitor concentration.

Kinetic parameters for uptake were obtained using the Michaelis-Menten Eq. (2);

2 $$v=\frac{{V}_{{\max }}\times [C]}{{K}_{m}+[C]}$$

where *v, V*_*max*_, *K*_*m*_, and [C] are initial uptake rate, maximal uptake rate, Michaelis constant, and substrate concentration, respectively.

*K*_*i*_ of crizotinib was obtained from the following Eq. (3);

3 $${K}_{i}=\frac{{V}_{{\max },app}}{{V}_{{\max }}-{V}_{{\max },app}}\times [{\rm{I}}]$$

where *V*_max,app_ is the apparent maximal uptake rate in the presence of inhibitor.

The unbound renal proximal tubule epithelial cell-to-medium concentration ratio (*K*_*p,uu*_) based on the steady-state uptake at 37 °C and 4 °C was defined according to Eq. (4) ^32^;

4 $${K}_{p,uu}=\frac{C/Mrati{o}_{ss,{37}^{^\circ }{\rm{C}}}}{C/Mrati{o}_{ss,{4}^{^\circ }{\rm{C}}}}$$

The C/M ratio_ss_ was calculated by subtracting bound compound after uptake for 1 min at 4 °C from the uptake for 120 min at 37 °C or 4 °C by rat kidney slices. *K*_*p,uu*_ was calculated by dividing $C/Mrati{o}_{ss,{37}^{^\circ }{\rm{C}}}$ by the mean value of $C/Mrati{o}_{ss,{4}^{^\circ }{\rm{C}}}$.

Model-based increase of SCr by TKIs was estimated according to Eqs (5 and 6) ^10^;

5 $${\rm{Increase}}\,{\rm{of}}\,{\rm{SCr}}( \% )=({{\rm{CL}}}_{{\rm{cre}}}^{\prime} /{{\rm{CL}}}_{{\rm{cre}}}\mbox{--}1)\times 100$$

6 $${{\rm{CL}}}_{{\rm{cre}}}=(1-{\rm{FR}})\times [{\rm{GFR}}+\tfrac{\tfrac{{\rm{RPF}}\times {{\rm{CL}}}_{{\rm{rs}},{\rm{int}},{\rm{all}}}}{(1+{{\rm{C}}}_{{\rm{\max }},{\rm{u}}}/\,{I}{{C}}_{50,{OCT}2}{\rm{or}}{{K}}_{{i},{OCT}2})\times (1+{{C}}_{{\rm{\max }},{\rm{u}}}\,/\,({K}_{p,uu}\times I{C}_{50,MATE1}{\rm{or}}{K}_{i,MATE1}))}}{{\rm{RPF}}+\tfrac{{{\rm{CL}}}_{{\rm{rs}},{\rm{int}},{\rm{all}}}}{(1+{{\rm{C}}}_{{\rm{\max }},{\rm{u}}}/\,I{C}_{50,OCT2}{\rm{or}}{K}_{i,OCT2})\times (1+{{\rm{C}}}_{{\rm{\max }},{\rm{u}}}\times {K}_{p,uu}\,/\,(I{C}_{50,MATE1}{\rm{or}}{K}_{i,MATE1}))}}],$$

where CL_cre_, CL_cre_′ FR, RPF, and CL_rs,int,all_ represent creatinine clearance, CL_cre_ in the presence of creatinine transporter inhibitor, the fraction reabsorbed, renal plasma flow, and the intrinsic clearance by tubular secretion, respectively. In this model, the intrinsic clearances of OCT2 and MATE1 were assumed to be equal. When MATE1 inhibition was predicted from the C_max,u_ of drugs, *K*_*p,uu*_ was assumed to be 1. For the prediction of CL_cre_ change, RPF was set as 545 mL/min/1.73 m^2^ according to Berg *et al*.^33^, and individual parameters CL_cre_, FR, GFR, and CL_rs,int,all_ of 11 subjects were taken from the reported clinical study^10^.

## Electronic supplementary material

SUPPLEMENTARY INFORMATION
