# Supplementary material for: Effect of tyrosine kinase inhibitors on renal handling of creatinine by MATE1
Source: Sci Rep. 2018 Jun 18;8:9237. doi: 10.1038/s41598-018-27672-y (PMC6006426; doi:10.1038/s41598-018-27672-y)
Supplement: Supplementary file 1 — SUPPLEMENTARY INFORMATION [file 41598_2018_27672_MOESM1_ESM.pdf]

SUPPLEMENTARY INFORMATION

Title: Effect of tyrosine kinase inhibitors on renal handling of creatinine by MATE1

Saki Omote[1], Natsumi Matsuoka[1], Hiroshi Arakawa[1], Takeo Nakanishi[1], and Ikumi Tamai[1,*]

[1]Faculty of Pharmaceutical Science, Institute of Medical, Pharmaceutical and Health Sciences, Kanazawa University, Kakuma-machi, Kanazawa 920-1192, Japan

* To whom correspondence should be addressed:

Department of Membrane Transport and Biopharmaceutics, Faculty of Pharmaceutical Sciences, Kakuma-machi, Kanazawa, Ishikawa, 920-1192, Japan,

Tel: +81-76-234-4479, Fax: +81-76-264-6284,

E-mail: tamai@p.kanazawa-u.ac.jp

Running Title:

Inhibition of creatinine transporter MATE1 by TKIs

Key Words

creatinine, tyrosine kinase inhibitor, kidney, OCT2, MATE1, secretion, transporter

Abbreviations

OCT2: organic cation transporter 2, MATE1: multidrug and toxin extrusion 1

TKIs: tyrosine kinase inhibitors, SCr: serum creatinine concentration

Supplemental Method

HEK293 cells transfected with human MATE2K (HEK293/MATE2K) and vector alone (mock) were gifts from Dr. Inoue (Tokyo University of Pharmacy and Life Science). The uptake study was conducted according to the same procedure as described in the main text.

Supplemental Results

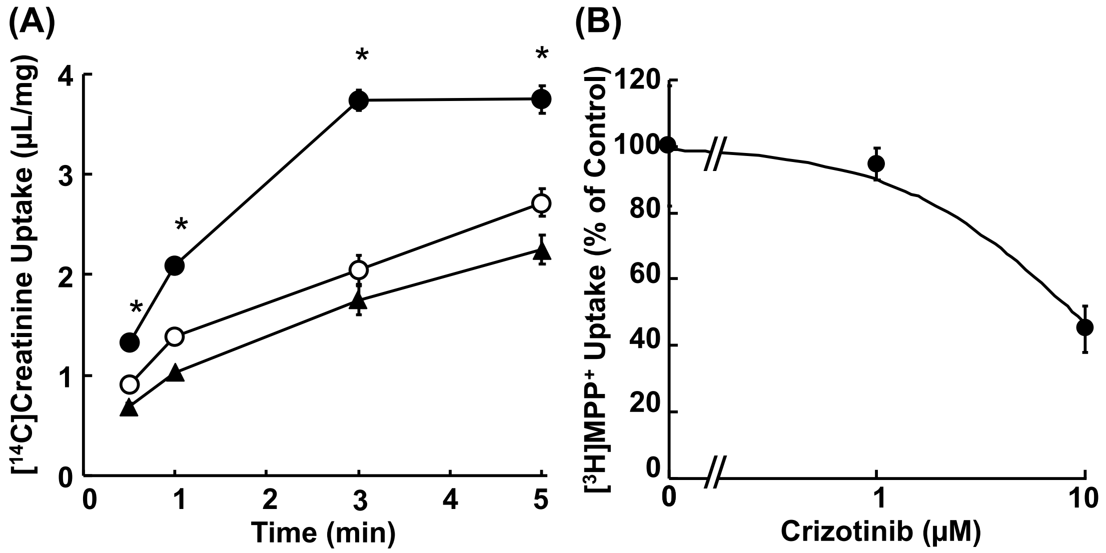

Supplemental Figure 1. Time-dependent uptake of [^{14}C]creatinine uptake by HEK293/MATE1 cells. (A) Uptake of [^{14}C]creatinine (2.6 µM) was measured for 0.5, 1, 3, and 5 min in HEK293/MATE1 (closed circles), MATE2K (closed triangle), and mock (open circles) cells. Each point indicates the mean of 3 determinations. Bars indicate ± S.E.M. (n = 3), and if not shown, are smaller than the symbol. * indicates a significant difference from mock cells (p<0.05) by Student t-test. (B) Uptake of [^{3}H]MPP^{+} (2.5 nM) was measured in the presence of crizotinib of 0, 1, and 10 µM for 1 min. Each point indicates the mean of 3 determinations. Bars indicate ± S.E.M. (n = 3).

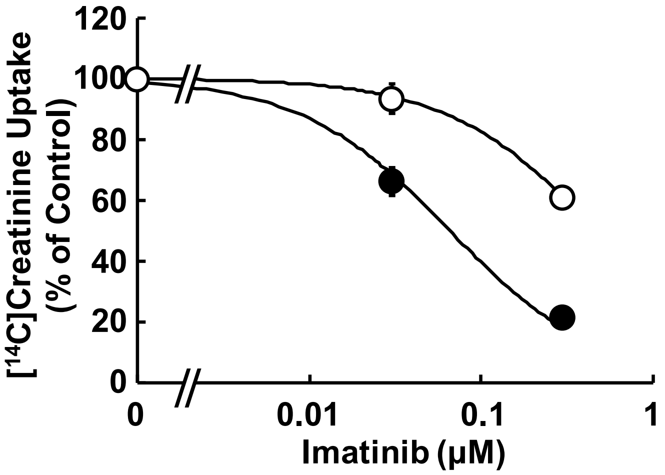

Supplemental Figure 2. Influence of preincubation on the inhibitory effect of imatinib on [14C]creatinine uptake by HEK293/MATE1 cells. The cells were pre/co-incubated with or without imatinib for 60 min, and then MATE1-mediated uptake of [14C]creatinine (2.5 μM) was measured for 2 min in HEK293/MATE1 cells. Open and closed circles represent co-, and pre/co-incubation with imatinib, respectively. Each point indicates the mean of 3 determinations. Bars indicate ± S.E.M. (n = 3), and if not shown, are smaller than the symbol.
